# Span of Supervision and Repercussions of Envy: The Moderating Role of Meaningful Work

**DOI:** 10.3389/fpsyg.2021.774688

**Published:** 2022-01-04

**Authors:** Hafiz Muhammad Burhan Tariq, Asif Mahmood, Ayyaz Ahmad, Maria Khan, Shah Ali Murtaza, Asif Arshad Ali, Edina Molnár

**Affiliations:** ^1^Institute of Business and Management, University of Engineering and Technology, Lahore, Pakistan; ^2^Department of Business Studies, Namal Institute, Mianwali, Pakistan; ^3^Institute of Quality and Technology Management, University of the Punjab, Lahore, Pakistan; ^4^Institute of Management and Organizational Sciences, University of Debrecen, Debrecen, Hungary

**Keywords:** envy, supervision, meaningful work, resource depletion, instigated incivility, work engagement, fast food

## Abstract

Though the current research stream has provided some risk factors for envy at the workplace, little is still known about the drivers and consequences of envy. Based on Vecchio’s theory, this study investigates the ripple effect of the span of supervision on envy. Moreover, it sheds light on the moderating role of meaningful work in their relationship. The data comprising sample size 439 were collected from confrères of four fast food companies listed on the Stock Exchange of Pakistan. Partial Least Square Structural Equation Modeling (PLS-SEM) technique was implemented through SmartPLS 3.3.2 to analyze the measurement and structural relationships. The results demonstrate that a narrow span of supervision will increase work engagement, and reduce instigated incivility via decreasing envy and resource depletion in sequence. Moreover, meaningful work would help regulate the inimical stream of dénouement of envy. Theoretical and practical implications, along with the limitations and future directions, have also been discussed.

## Introduction

A wider span of supervision restricts the honcho’s prowess to facilitate the subordinates in various aspects, such as emotional, instrumental, appraisal, and informational (Mueller, 2012). It is because a wider span of supervision, either through downsizing or increased centralization, puts a leader under enormous strain and expectations (Thompson and Li, 2010). This increased pressure may refrain a leader from supporting the followers when required. As a result, a wider span of supervision can goad the confrères into instigated incivility or work disengagement (Thompson et al., 2016). Similarly, the scarcity and competition for resources produce high levels of envy. Envy is a universal emotion aroused by another’s good fortune (Li et al., 2021) that damages relationships because it can result in spiteful behavior (González-Navarro et al., 2018). Since envy and depletion could direct employees to take the emotional direction (Tandoc et al., 2015), it is vital to control their emotions to better an organization and enhance work engagement.

Envy is an emotion that people experience when they think someone is better than them (Van de Ven, 2017). Traditionally, it could be construed as painful and annoying emotions because of inferiority feelings, facing antipathy behavior, and hostility (Kim et al., 2010). Besides this, envy is related to personal response-dependent variables like behavior and affective response (Puranik et al., 2019). In turn, behavior and affective response consist of actors such as a sense of rejection, distress, resentment, job dissatisfaction, anger, and fear (Lee et al., 2018). For, envy is a painful emotion by definition, therefore, employees deplete their self-regulatory resources to overcome this traumatic emotion (Christian et al., 2014). Hence, employees will be unable to make use of their full energy at the workplace. Thus, it can be concluded from this description that envy is a source of harmful or hostile behavior at the workplace. However, envy can also yield positive consequences, such as motivating increased performance or attempts at self-improvement. These contradictory understandings illustrate that the study of envy and its work-related outcomes have been surprisingly sparse (Shu and Lazatkhan, 2017). Shortly, there are two dimensions of envy: benign envy (motivate the envier to strive toward greater heights and causes people to invest more effort to be as successful as the other person); and malicious envy (envier aims to harm the envied and motivates people to level the other person down) (de Zoysa et al., 2021). The traditional view of envy at the workplace focuses on malicious envy (Shu and Lazatkhan, 2017). Envy can be categorized into three forms: envy others in a work setting (Vecchio, 1995; Duffy and Shaw, 2000); temperamental envy across all settings (Smith and Kim, 2007); and spasmodic envy associated with a particular person (Cohen-Charash, 2009). However, in this study, we are building our premise on the first type of envy in which an individual worker inimically compares himself or herself with the confrères.

Different studies have discussed antecedents and consequences of envy, but the broad conceptual perspective is lacking (Li et al., 2021). To address the dilemma that most current managers/leaders face in managing the emotions of subordinates, the current state of the research on envy is not informative enough (González-Navarro et al., 2018), and it is essential to fill this gap to understand the dynamics underlying the relationship between envy and counterproductive work behavior. Workplace envy significantly predicts counterproductive behaviors and organizational citizenship behavior (Ghadi, 2018). Yu et al. (2018) have investigated employees’ downward envy for making supervisors abusive via threat to self-esteem of supervisor. Similarly, Demirtas et al. (2015) found that envy is positively linked with the depletion of resources. Moreover, envy has effects on social undermining via moral disengagement (Duffy et al., 2012), and engagement at work may reduce because of envy (Demirtas et al., 2015) as workplace envy is negatively associated with engagement (Li et al., 2021). Moreover, Mao et al. (2020) have discussed that envy is positively related to incivility. However, literature is far from the relevant variables of the competitive reward structure and meaningful work included in Vecchio’s theory when examining the antecedents of envy (Thompson et al., 2016). Furthermore, the literature neglects the mechanism of envy: how the span of supervision predicts the association with work engagement via envy and depletion of resources in sequence, and how moderating role of meaningful work help break the domino effect of envious behavior.

Building on this body of research, this study contributes to the literature by advancing our understanding of the cause-effect relations regarding envy. First, we examine the span of supervision as a cause of envy. Given that managerial practices (e.g., meaningful work and compensation systems) substantially influence employees’ lives in an organization and leaders’ differential treatment of employees may induce unfavorable social comparisons that promote feelings of envy. Second, this research seeks to understand the varying consequences of envy. Existing research has focused on understanding the direct effect of envy on various behavioral outcomes (e.g., work engagement and instigated incivility). Mechanisms involved in producing behavioral effects of envy are not much clear. Researchers have called for more research to examine the other variable linking envy with behavioral outcomes. For example, mediating variables can clarify the underlying process of envy affecting employees’ behaviors (Ghadi, 2018). To date, this research is in a nascent stage, with relatively few researchers directly exploring the mechanisms connecting envy to outcomes (Duffy et al., 2021). Responding to the call of researchers to examine the mechanism between envy–outcome relationships, resource depletion is presented as a potential mechanism in understanding the linkage between envy and work engagement and instigated incivility. The present study aims to examine the role of envy at work as a mediator between a set of antecedents and consequences. It also analyses meaningful work as a moderator between the span of supervision and envy; also the role of mediator between envy and consequences as recommended by Ghadi (2018) that it would be noteworthy for future studies to extend his hypothesized model by including mediating and moderating variables to clarify the underlying process by which envy affects employees’ behaviors.

Based on this frame of reference, the theoretical framework of the study was developed. From the work unit, the span of control variable was selected as a predictor, while resource depletion was chosen as a reasonable response of envy, and finally, work engagement was selected as the behavioral response. Moreover, with the further development in envy, it has been argued that meaningful work can be used as a moderator to reduce envy. Through SEM using the Smart partial least square (PLS) software, statistical verification analysis was done in this research.

Theoretically, this research contributes in multiple ways. It enriches the literature by suggesting that the supervisory span is linked to incivility by a process that sequentially reduces envy and resources. This research proposes that if the span of supervision is significantly more, employees may engage in destructive behavior at the workplace, such as paying less attention to work and embroiling in uncivil behavior as the instigator. In this context, this is the first study that links the span of supervision to the work engagement mechanism. Moreover, this study is essential for all honchos as it highlights the factors which cause an increase in organizational, operational costs. Furthermore, this research is drawn on Vecchio’s (1997) theory to test the impact of the span of supervision on work engagement via two mediations— envy and depletion of resources— in sequence.

Vecchio (1997) developed a theory that indicates three independent sets of variables that influence envy. First is “individual differences,” which consist of work ethics, in-group status, dependency, gender, and self-monitoring. The second is “work unit,” which composes of supervisory differentiation of attitudes, job rotation, unit size, supervisory considerations, and reward system. The third is “national culture attributes,” which comprise collective norms, cooperative norms, employee participation norms. The theory further elaborates that department heads are institutions that regulate employees’ natural envy phenomenon. Thus, a supervisor can improve or reduce envy among employees using supportive or non-supporting conduct. Moreover, differentiation of control actions may enhance competition in the workforce among workers.

The article is organized into five sections: first, the literature on workplace envy and the theoretical background of envy at work are reviewed. The following section deals with methods used to analyze data. After that, the empirical results of the theoretical model are presented. Finally, this article concludes with a discussion of the findings and implications and future research recommendations with limitations.

## Literature Review

### Inclusion Criteria

In November 2020, we searched the following databases: Web of Science, PsycINFO, EBSCO, and Theses Global. “Envy” was used as the keyword. We also searched the Academy of Management 2009–2020 to identify all relevant published or unpublished empirical studies. Considering our inclusion criteria (empirical studies that measured envy with quantitative statistics), initially, we retrieved 965 sources containing articles, dissertations, and unpublished data. We narrowed the database pool by excluding any irrelevant research or lacking the necessary statistical information, like sample size (N) and correlation coefficient (r). This reduced the sample to 69 sources. The sample was reduced to 47 sources after eliminating studies that did not provide the key variables for a relationship of interest and an appropriate theoretical model.

### Hypotheses Development

Building on the available literature, this section develops theoretical justifications of the relationships between antecedents and consequences of workplace envy.

### The Span of Supervision and Envy

According to Vecchio’s (1997) theory, the span of supervision is positively associated with envy because a supervisor can reduce envy among his/her followers by providing leadership to their followers. Conversely, a supervisor may enhance envy among followers if he has a different relationship with the followers. The reason is that followers who think their supervisor is closer to other followers enhance envy among followers who have not such a relationship. Supervisors increase the covetous to give promotion to some employees with whom they have a close relationship on the one hand (Vecchio, 1997). While on the other hand, followers who receive such malicious behavior compared to other employees may enhance envy among them (Kim et al., 2010). So, from this description, it can be concluded that the span of supervision positively predicts envy. Considering the above discussion, the current research study proposes the following hypothesis:

H_1_: Span of supervision is positively associated with envy.

### The Span of Supervision and Resource Depletion via Envy

Lange et al. (2018) indicate that envy consists of three factors, malicious, pain, and benign envy. Moreover, envy is positively associated with schadenfreude in malicious form and not with the other two forms. In general, the researchers in the literature have studied that organizations are facing problems because of envy.

Though the span of supervision is positively associated with envy, it is predicted that the span of supervision may trigger resource depletion among employees. Here, resources represent self-control which aligns with energy and attention (Lilius, 2012). Self-regulate resources are to devote full energy at the workplace in a positive way; hence these contribute to performance (Thau and Mitchell, 2010). Empirically, these types of help may replenish or deplete because of interpersonal events at the workplace (Bono et al., 2013).

It is suggested in this study that resources deplete as a result of envy (Koopman et al., 2020; Duffy et al., 2021), is a consequence of a wider span of supervision (Thompson et al., 2016). The basis of this assumption is envy. Vecchio (1997) represented this assumption by indicating that the span of supervision is a predictor of envy, and resource depletion is an adequate response. Koopman et al. (2020) conducted a survey study to test this hypothesis, and the result indicated a positive relationship between envy and resource depletion. This is because envy is painful (Tai et al., 2012), and to overcome this pain, employees deplete their self-regulatory resources (Christian et al., 2014). Moreover, Thompson et al. (2016) tested the relationship between the span of supervision and envy, and the result provided a positive association between the span of supervision and envy. So, from all this evidence, it is assumed that the span of supervision positively impacts resource depletion via envy. Considering the above discussion, the current research study propounds the following hypothesis:

H_2_: Span of supervision is positively associated with resource depletion, via envy.

### Span of Supervision and Work Engagement via Envy and Resource Depletion

Having surmised that the span of supervision is positively linked with resource depletion via envy, we propose that work engagement wanes by a wider span of supervision. Work engagement is defined as a relatively enduring state of mind, referring to the simultaneous investment of personal energies in work performance (Yam et al., 2018). Research indicates that work engagement is connected with physical energy, emotions, and cognition (Rich et al., 2010). Empirical evidence provides that work engagement may also be linked with leader behavior (Yam et al., 2018). It is, therefore, postulated here that dwindled work engagement is a dénouement of depletion of resources (Weiss et al., 2018), resulting from envy (Koopman et al., 2020), and triggered by the span of supervision (Thompson et al., 2016). In other words, envy leads to an unpleasant mood and anxiety that reduces work engagement and performance (Lee et al., 2018). Theoretically, from Vecchio’s (1997) point of view, this postulate can be described as that span of supervision may be used as a predictor of envy. As employees think that supervisors give less attention to them than some other workers, it whips up envy among such employees. This envy leads toward depletion of resources among employees as a compelling response to this envy. Hence, this depletion of resources leads to less work engagement as a behavioral response from the depletion of resources. Empirically, there is an eventual obliteration from the span of supervision to work engagement. So, from all this detail, it can be concluded that the span of supervision negatively predicts work engagement via envy and depletion of resources in sequence. Considering the above discussion, the current research study proposes the following hypotheses:

H_3_: Span of supervision is negatively associated with work engagement via envy and resource depletion in sequence.

H_4_: Resource depletion is negatively associated with work engagement.

### Span of Supervision and Instigated Incivility via Envy and Resource Depletion

Employees are likely to use counterproductive work behavior when they feel envy at the workplace, but such behaviors may differ depending on the ownership of organizations (González-Navarro et al., 2018). Granted that span of supervision is negatively associated with envy and depletion of resources in sequence, we are also propounding here that span of supervision is likely to enhance instigated incivility. Instigated incivility is one type of incivility; the other two beings witness incivility and experienced incivility. Instigated incivility is opposite to experienced incivility. In a broader term, incivility by nature is instigated because incivility encourages spiral negativity in the workplace due to mutual benefits (Schilpzand et al., 2016). Here, we contend that instigated incivility stems from a span of supervision (Thompson et al., 2016) due to the depletion of resources (Koopman et al., 2020). In other words, a span of supervision can be used as a predictor of envy, and depletion of resources is a compulsive response of envy that leads to behavioral riposte such as instigated incivility (Vecchio, 1997). On a rational footing— from the span of supervision to instigated incivility— it is posited that the supervisor cannot provide time and support to every employee because of time constraints. The employees who are unable to get the supervisor’s attention get engaged in instigated incivility by backbiting their colleagues as proved by Mao et al. (2020) that envy is positively related to the incivility of employees toward coworkers. The relationship between resource depletion and instigated incivility has been tested by Thompson et al. (2016) and found a positive relationship between these two variables. So, from all this description, it can be concluded that span of supervision positively predicts instigated incivility via envy and resource depletion in sequence. Considering the above discussion, the present research study put forward the following hypotheses:

H_5_: Span of supervision is positively associated with instigated incivility via envy and resource depletion in sequence.

H_6_: Resource depletion is positively associated with instigated incivility.

### Meaningful Work as Moderator in the Relationship Between Span of Supervision and Envy

In addition to the direct effect of the span of supervision on envy, this study also intends to examine meaningful work as a moderating factor in understanding the linkages between these two variables of the study. At this point, we argue that meaningful work reduces the envy element among employees caused by a wider span of supervision. Meaningful work can be characterized by the state when employees think that their work has a positive significance (Demirtas et al., 2015). When employees perceive the importance of their work, they put their cognitive resources, pay full attention and energy to that work (Rosso et al., 2010). In this sense, it is assumed that employees do not pay heed to the factors such as a wider span of supervision, and only be attentive toward their work. From this point of view, it can be concluded that when there is a perception of meaningful work, the employees do not think about the span of supervision, which is a predictor of envy. In this way, there is less vehemence of envy among employees, which leads them to utilize their resources for work performance behavior such as work engagement. Considering the above discussion, we put forward the following hypothesis:

H_7_: The positive relationship between the span of supervision and envy is moderated by meaningful work such that the relationship is weaker when there is high knowledge about meaningful work, and strong when there is a low level of knowledge about meaningful work.

**FIGURE 1:**
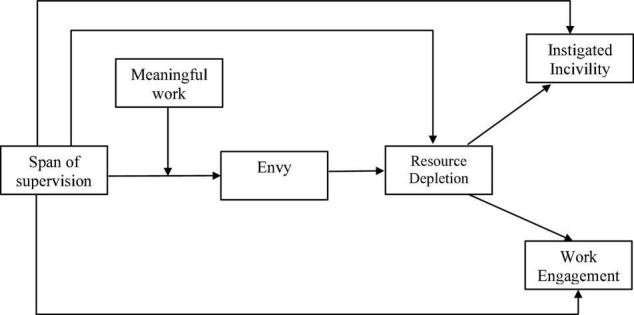
The hypothesized model.

Encapsulating all the relationships, the theoretical can be outlined as shown in Figure 1.

## Materials and Methods

### Research Design

To empirically test the proposed hypotheses, a statistical technique “Structural Equation Modeling” (SEM) using Partial Least Squares (PLS) was used as the structural model is complex and includes many constructs, indicators, and/or model relationships. PLS-SEM is recommended when the analysis is concerned with testing a theoretical framework from a prediction perspective, primarily used for exploratory research and when distribution issues are a concern, such as lack of normality (Hair et al., 2019). In contrast to AMOS-SEM, the statistical objective of PLS-SEM is to maximize the variance explained in the dependent variable(s), and it is based on the composite model. Also, PLS-SEM achieves greater statistical power at all sample sizes (Hair et al., 2017).

### Respondents and Procedure

The study was conducted on employees working in four different food organizations listed with Pakistan Stock Exchange, located in Pakistan. Such organizations have to serve their customers better and, thus, require their employees to be engaged. Adopting a cross-sectional design, a self-report questionnaire was used to collect data from Burger King, Fat Burger, KFC, and McDonald’s, and a convenient sampling technique was used. The condition for participating in the study was to be currently working. In all, 689 respondents were approached for taking data, but the present study is based on 439 respondents (63.74% response rate). Some of the remaining questionnaires were not returned by employees and some were not usable because of the incomplete filled-in questionnaires. Data were gathered from employees working at various organizational levels. The researcher approached respondents personally. Both genders were included in the study sample, and the majority were male (78% approximately). The age of respondents ranged from 20 to 40 years, with the average being 24 years approximately. The minimum qualification of the study sample was intermediate. Respondents were provided with all the necessary information such as study objectives, methods of responding to the questionnaire, etc. Respondents were also assured that their responses would be kept confidential and that there were no right or wrong answers. Table 1 indicates the values of descriptive statistics for the 439 respondents of the research study.

**TABLE 1:** Demographics of the respondents.

Items	Frequency	Percentage
**Marital status:**		
Divorced	16	3.7
Married	126	28.7
Single	297	67.6
**Gender:**		
Female	97	21.9
Male	342	78.1
**Age:**		
20–25	233	53.01
26–30	90	20.47
31–35	83	18.89
36–40	33	7.63
**Education:**		
12 Years	86	19.7
14 Years	229	52.1
16 and Above	124	28.3
**Experience:**		
1 and Less	116	26.3
2–5	289	65.9
6–10	34	7.8

### Measures

Already established scales were used for measuring the response of the participants, as reported in the Supplementary Appendix. All the items were listed on a five-point Likert scale (1 = strongly disagree and 5 = strongly agree). The single item scale of the span of supervision has been taken from Thompson et al. (2016). The only item reads, “Leaders reported the number of their subordinates in each workgroup.” The reliability of the scale was not checked due to the single-item construct. Envy was measured by using five items taken from Thompson et al. (2016). The sample item is “My Supervisor values the efforts of others more than she/he values my efforts.” The reported Cronbach’s alpha value was 0.86 of these five items. Twenty-six items of the resource depletion scale from Christian and Ellis (2011) were used to capture the response of the participants. The sample item is “I feel mentally exhausted.” The alpha value was 0.96. The scale for work engagement was adopted from Yam et al. (2018) to measure the response of the respondents. The total number of items on this scale is eighteen. A sample item is “I work with intensity on my job.” The alpha value for this scale was 0.91. Instigated incivility was measured with four items from Rosen et al. (2016). An example item is “Put you down or was condescending to you.” Cronbach’s alpha value for this scale was 0.87. Meaningful work was assessed by ten items (Steger et al., 2012). An example item is “I understand how my work contributes to my life’s meaning.” The stated alpha value was 0.92.

## Data Analysis and Results

The analysis has been conducted through Partial Least Square-based Structural Equation Modeling (PLS-SEM) with the help of SmartPLS 3.2.2. The prime reason for applying this technique is the presence of a single item construct “span of supervision.”

### Measurement Model

The reliability assessment of the reflective constructs employed in the study was undertaken by Cronbach’s alpha and composite reliability (Hair et al., 2011). The acceptable threshold value is 0.70, whereas, in exploratory research, the value greater than 0.60 is also adequate (Raykov, 2007). Table 2 shows the results of the reliability test using SmartPLS for the variables— Span of Supervision, Envy, Resource Depletion, Work Engagement, Instigated Incivility, and Meaningful Work.

**TABLE 2:** Outer loadings, Cronbach’s alpha and AVE.

Variable	Symbols	Loadings	Cronbach’s Alpha	Average Variance Extracted (AVE)
Envy (En)	Envy1	0.827	0.935	0.795
	Envy2	0.899		
	Envy3	0.918		
	Envy4	0.905		
	Envy5	0.905		
Instigated incivility (II)	Instigate1	0.851	0.924	0.815
	Instigate2	0.921		
	Instigate3	0.919		
	Instigate4	0.918		
Meaningful work (MW)	Mean_1	0.821	0.959	0.730
	Mean_2	0.879		
	Mean_3	0.824		
	Mean_4	0.862		
	Mean_5	0.827		
	Mean_6	0.859		
	Mean_7	0.834		
	Mean_8	0.873		
	Mean_9	0.861		
	Mean_10	0.903		
Resource depletion (RD)	ResDep1	0.709	0.961	0.539
	ResDep2	0.717		
	ResDep3	0.774		
	ResDep4	0.745		
	ResDep6	0.726		
	ResDep7	0.714		
	ResDep8	0.736		
	ResDep9	0.753		
	ResDep10	0.713		
	ResDep11	0.756		
	ResDep12	0.711		
	ResDep13	0.768		
	ResDep14	0.707		
	ResDep15	0.756		
	ResDep16	0.702		
	ResDep17	0.730		
	ResDep18	0.721		
	ResDep19	0.745		
	ResDep20	0.702		
	ResDep21	0.794		
	ResDep22	0.735		
	ResDep23	0.751		
	ResDep24	0.715		
Span of supervision (SS)	Span	1.000	1.000	1.000
Work engagement (WE)	Work1	0.741	0.949	0.564
	Work3	0.730		
	Work4	0.762		
	Work5	0.782		
	Work6	0.745		
	Work7	0.744		
	Work8	0.737		
	Work9	0.779		
	Work10	0.708		
	Work11	0.785		
	Work12	0.737		
	Work13	0.717		
	Work14	0.779		
	Work15	0.771		
	Work16	0.769		
	Work17	0.719		

It can be observed that the Cronbach’s alpha values of all the constructs are greater than the threshold of 0.7. The Cronbach’s alpha values for Envy, Instigated Incivility, Meaningful Work, Resource Depletion, Work Engagement are 0.935, 0.924, 0.959, 0.961, and 0.949, respectively, while Span of Supervision has a value of 1.00 because it is a single item construct.

The construct validity was established through convergent and discriminant validities. The convergent validity assesses whether a particular construct is measuring that construct. It is evaluated through outer loadings and Average Variance Extracted (AVE) for each variable under consideration. The suggested acceptable value of AVE is >0.50, which indicates 50 percent of the variance in the variable is because of explaining indicators of the variable (Henseler et al., 2014). Similarly, the threshold value for outer loadings is 0.7. It can be seen that the values of loadings for all the constructs are greater than 0.7. Likewise, Table 2 also reports AVE values for each construct. It can be noticed that AVE values for all the constructs are greater than the threshold value. Consequently, the construct validity of the scale has been established. On the other hand, discriminant or divergent validity gauges the independence of one measure from the other measures of the same construct (Drost, 2011). The criterion defined by Fornell and Larcker (1981) has been used to assess the discriminant validity, which states that the square root of the AVE value for each construct should be greater than all its correlations with other constructs.

Table 3 shows the results of discriminant validity for Envy, Instigated Incivility, Meaningful Work, Resource Depletion, Span of Supervision, and Work Engagement. The diagonal values indicating the square roots of AVE for all the constructs are higher than their corresponding correlations. Hence, discriminant validity has also been established for the scale.

**TABLE 3:** Fornell-Larcker criterion.

	En	II	MW	ME	RD	SS	WE
En	0.891						
II	0.143	0.903					
MW	–0.339	–0.089	0.855				
ME	–0.336	–0.022	0.328	1.000			
RD	0.319	0.298	–0.076	–0.029	0.734		
SS	0.466	0.226	–0.215	–0.381	0.267	1.000	
WE	–0.130	–0.030	0.017	0.057	–0.202	–0.227	0.751

### Structural Model Analysis (Hypotheses Testing)

**FIGURE 2:**
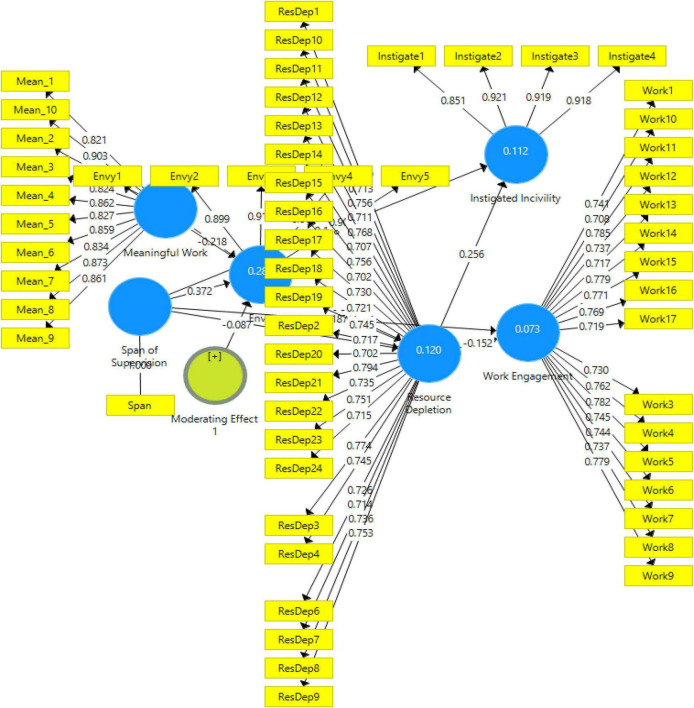
Structural equation model.

The association among the latent variables is examined by evaluating the inner model once after the satisfactory results of validity and reliability (Garson, 2016). The reason for the inner model assessment is to check the path coefficients, and how much variance is explained in the endogenous variable by the exogenous variable within the model under study. SmartPLS, by its built-in analysis, give multiple criteria to examine the inner model (Wong, 2013). This analysis includes path coefficients (beta values), t-statistics, and *p*-values for each path. The *p*-values have been estimated by adopting bootstrapping procedure. Figure 2 shows the output of the structural equation model, whereas Tables 4, 5 summarize the path coefficient values along with t-statistic and *p*-values for each path.

**TABLE 4:** Direct effects.

Direct effects	Path coefficients	T statistic	*P* values
Envy – > Resource Depletion	0.248	5.932	0.000
Resource Depletion– > Instigated Incivility	0.256	6.125	0.000
Resource Depletion – > Work Engagement	(−0.153	3.718	0.000
Span of Supervision – > Envy	0.375	7.484	0.000
Span of Supervision– > Instigated Incivility	0.157	4.456	0.000
Span of Supervision– > Resource Depletion	0.154	3.549	0.000
Span of Supervision – > Work Engagement	−0.191	4.404	0.000

**TABLE 5:** Mediation analysis (indirect effects).

Mediation effects	Path coefficients	T statistic	*P* values
SS – > En – > RD	0.093	5.022	0.000
SS – > En – > RD – > WE	−0.014	2.863	0.004
SS – > En– > RD – > II	0.024	3.779	0.000

Table 4 indicates that all the direct paths are substantial and statistically significant. For instance, the path from Envy to Resource Depletion has a beta value of 0.248, which is positive and significant with a *p*-value of 0.000 and a t-statistic value of 5.932.

On the other hand, Table 5 shows the indirect effects for three mediations; one is single while two are double mediations in sequence. The indirect impact of Span of Supervision on Resource depletion via Envy is positive and significant with coefficient value = 0.093 and *p*-value = 0.000. Considering the results, it is proved that the Span of Supervision is Positively associated with Resource Depletion via Envy. Similarly, the indirect effect of Span of Supervision on Work Engagement via Envy and Resource depletion is negative and significant with coefficient value = −0.014 and *p*-value = 0.004. In the light of the above results, it is confirmed that Span of Supervision is negatively associated with Work Engagement via Envy and Resource Depletion. While the indirect effect of Span of Supervision on Instigated Incivility via Envy and Resource depletion is positive and significant with coefficient value = 0.024 and *p*-value = 0.000. Taking into account the results, it is verified that the Span of Supervision is positively associated with Instigated Incivility via Envy and Resource Depletion.

Although the magnitude of mediations is smaller, they still show significant effects. These meager indirect effects have been reinforced by introducing Meaningful Work as a moderator to offset the inimical positive effect between Span of Supervision and Envy. Table 6 exhibits that the direct relationship between Span of supervision and Envy is positive and significant; the link between Meaningful Work and Envy is negatively significant.

**TABLE 6:** Moderation effects.

Moderation effects	Sample mean	T statistics (|O/STDEV|)	*P* values
Meaningful Work – > Envy	−0.214	4.076	0.000
Moderating Effect (SS × MW) – > Envy	−0.088	2.129	0.034
Span of Supervision – ( Envy	0.375	7.484	0.000

**FIGURE 3:**
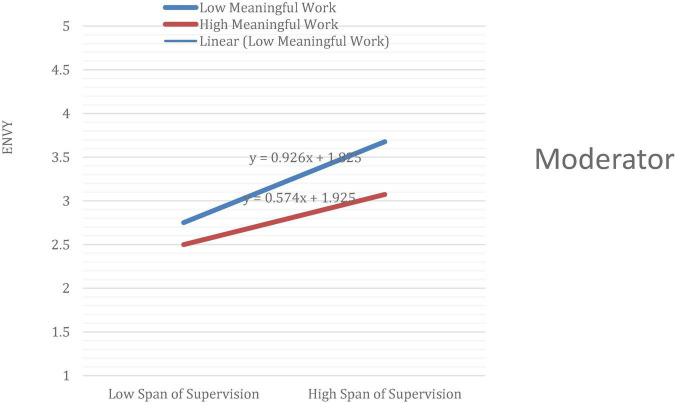
The moderation graph.

Moreover, the interaction term (moderating effect) of Meaningful Work on the relationship of Span of supervision and envy is negatively significant. It means that meaningful work moderates the relationship between the span of supervision and Envy. In other words, the relationship between the span of supervision and envy has been moderated by meaningful work such that the relationship weakens significantly when there is high knowledge about meaningful work and stronger when there is a low level of knowledge about meaningful work as depicted in Figure 3.

## Discussion

The theoretical model of workplace envy used in this study would provide a new foundation for studying the phenomenon of envy at the workplace. It was predicted that envy, referred to as pain at other’s good fortune, is a homeostatic feeling aroused by a wider span of supervision. The feeling of envy can stimulate both kinds of tendencies like threat-oriented and challenge-oriented. However, the current study has only focused on the threat-oriented approach. In self-control attempts due to the absence of a proper supervisory role, the employee shows the behavior of instigated incivility and less work engagement as a result of resource depletion. But meaningful work can minimize and even cast favorable effects of the span of supervision on envy by reversing the relationships.

This study implicates Vecchio’s theory, as hypothesis 1 predicts that span of supervision is associated with envy directly. Supervisors are entities who can control the natural phenomenon of envy among subordinates. A supervisor may enhance as well as reduce envy among confrères by using supportive or non-supportive behavior. Unfairness in supervisors’ conduct for their employees can be a reason to increase envy at a workplace (Vecchio, 1997). Favorable support only to some fellows can create envy among those receiving unfavorable behavior from their supervisor (Kim et al., 2010). While studying antecedents and consequences of Envy, Thompson et al. (2016) have suggested that a wider span of supervision would consequently limit the abilities of a supervisor. Failure in emotional support, informational support, and care to followers are the consequences of a large span of supervision. Hence, the current study supports Vecchio’s assumption that a span of supervision is a predictor of envy.

The “supernatural force,” which links the span of supervision with envy as its predictor, also triggers employee resource depletion. According to Hypothesis 2, resource depletion and span of supervision are interlinked positively via envy. Behavioral consequences of envy have their explanation in equity theory. Compared to other employees in the organization, people equate their outputs with their inputs in a ratio to assess equity (Adams, 1965). After such social comparison, if they find any inequality, they would feel the pain, which would lead them to envy (Heider, 1958). After feeling such pain and unfavorable comparison, they would try to minimize the pain in different ways to restore their equity (Pinder, 2008). Resource depletion, associated with vitality and energy, and considered self-control, can arise due to envy (Lilius, 2012). Koopman et al. (2020) also confirmed resource depletion among confrère as a result of envy. Therefore, in line with the previous studies, the span of supervision results in envy, which, in turn, strongly connects to resource depletion.

Whereas, Hypothesis 3 postulates that the span of supervision is negatively associated with work engagement through envy and resource depletion in sequence. If an open expression of envy at the workplace is not supported, it can put an employee in a more harmful situation. Therefore, people usually find alternate resources to restore equity with their targets (Schweitzer et al., 2006). Organizations can suffer due to this behavioral outcome of employees as a result of envy, which is further ignited by a larger span of supervision. There are alternate ways a person can react to this unfavorable situation. The comparison of outputs to inputs would cause an employee to be less productive to restore equity (Pinder, 2008). It may make a person less engaged in work-related activities. While in a result of social comparison relationship, the envious employee would impose the responsibility of such unfavorable behavior to the organization- the phenomenon explained in the past research of perceived injustice (Konovsky and Folger, 1991). Thus, the suggested postulate in this study is supported by the literature that the span of supervision via envy can decrease work engagement when resources depletion is sequenced.

Hypothesis 4 of the study suggests that resource depletion, as a behavioral outcome aroused by envy, would not give positive results when directly linked with work engagement. An envious employee would respond to the unfavorable situation by minimizing his positive contribution to the organization (Hannah-Moffat and Shaw, 2000). Supervisors can not pay equal attention to every employee in larger workgroups. Hence, they differentiate among them to address the essential matters. So, this kind of employee considers their supervisors, which leads other employees to envy, as suggested by the first hypothesis of the study. It could further lead to resource depletion, which means they sometimes lose control over the situation and may become low productive employees by fear of rejection or to restore their equity. By following Vecchio (1995), it can be assumed that an employee with a feeling of low self-esteem would make some response to regain his worth in the eyes of the supervisor. A counterproductive work behavior can detract him from work engagement to other malicious work activities. Liu et al. (2019) argued further that envy is a negative emotion that could be hostile to an organization, resulting in job turnover intention and absenteeism. Similarly, Leach et al. (2008) observed that many organizations are commonly facing the situation in which the workforce of their organization is experiencing adverse circumstances due to a large span of supervision. Hence, the results of hypothesis 4 are in line with the past studies.

Like many other studies, the current research has focused on the adverse effects of envy on organizations. Koopman et al. (2016) follows the routing path toward instigated incivility as envisaged in this study. Another negative work behavior among envious employees can be seen as instigated incivility through resource depletion. Instigated incivility is the contradiction of experienced incivility in which envied employees encourage to spread negativity in the workplace for personal gain or to gain the status of recognition among other employees (Schilpzand et al., 2016). Since the supervisor’s role is not enough in a large span of supervision, people who envy those who have close relationships with supervisors start to engage in civil activities like backbiting others (Thompson et al., 2016). Thus, the past research findings are consistent with hypothesis 5, which states that the span of supervision is associated with instigated incivility positively via envy and resource depletion in sequence. Similarly, hypothesis 6 is supported through the literature, which states that resource depletion is positively associated with incivility.

Lastly, hypothesis 7 of the current study suggests that in a meaningful work environment, the relationship of the span of supervision with envy can be mended. The past studies indicated that the span of supervision could create envy, which would give adverse outcomes. However, the perception of meaningful work may enable employees to understand the importance of their work and put all their cognitive resources and energies into work (Rosso et al., 2010). Hence, hypothesis 7, which is the contribution of the study, is indirectly supported by the previous outcomes.

## Theoretical and Managerial Implications

The present study is the validation of Vecchio’s theory in the food organizations of Pakistan listed with the stock exchange. The basis of the study lies in positivism, which beliefs in the logical mixing of data. Previous research literature has suggested the positive relationship between the span of supervision and envy by implicating Vecchio’s theory. Still, no study has indicated that how the span of supervision can be minimized. This research gap has been identified and bridged by using meaningful work as a moderator in the current study. The study would help supervisors minimize envy among the subordinates created by a wider span of supervision. If the depletion of resources produced by envy is addressed positively, then this problem can be solved before taking confrères to the instigated incivility level. Group leaders or supervisors can provide their support and aid to employees to repress the dénouements of envy, which is the source of resource depletion (Mueller, 2012). This approach can reduce envy among employees.

This study can be applied to all organizations because every organization has similar problems engendered by workplace envy. This study has importance for all levels of honchos. The direct supervisors should highlight the importance of meaningful work because this study has proven that meaningful work can minimize the adverse dénouements of envy. The current research suggests that the engagement and performance of employees can be enhanced by giving proper attention to understanding the importance of their work. The study also implicates the importance of depletion resources in the presence of a wider span of supervision. It has indirectly suggested that focusing on each employee by making subgroups and effectively may contract the destructive role of envy. Therefore, organizations should pay attention to minimizing the conflicts among confrères to control envy, and enhance work engagement for the betterment of organizations. Organizations should recognize that the toxic and destructive nature of emotion (envy) has a positive and constructive side and should not work on removing the triggers of envy; instead may be promoted and managed. Furthermore, insights generated from this study may be helpful for organizations to take steps to reap the benefits of envy experienced by employees adequately. Honchos should focus on meaningful work allocation and favorable resource distribution to the right people within the organizations. Furthermore, the proposed model of this study may provide managers with new insights into reducing envy at work.

## Conclusion

A theoretical framework for envy in workplace research has been provided by the analytical envy paradigm used in this analysis. Many studies have been conducted on Envy, but the information available is still scarce about its domino effect. This research is based on the principle of Vecchio, which assumes that supervisory span is related explicitly to envy. Supervisors are individuals who are capable of managing workers’ inherent jealousy. By utilizing positive or unsupportive behaviors, the honcho may enhance and evoke envy among confrères. Differentiating their employee’s supervisory actions, caused by a wider span of control, can increase their jealousy in the workplace (Vecchio, 1997). The results of the current research study align with a traditional view of envy as an unpleasant emotion that triggers negative or irrational behavior causing a detrimental conflict in the workplace and discussed several managerial and theoretical implications for the higher management of the fast-food industry of Pakistan. Following these guidelines, the honchos of the fast-food industry should pay more attention to the betterment of the performance of their employees to create a healthy organizational climate as the findings indicate that the span of supervision functions as an imperative antecedent of envy. Furthermore, envy at work has counterproductive consequences such as resource depletion.

The study assumed that envy or envying-others dimension, being a homeostatic emotion as a wider field of surveillance, can be named as pain for the good fortune of others. The sense of envy can stimulate both types of vehemence, such as threats and challenges. However, this study focused on a threat-oriented approach or dark side of envy that may lead to gloating or even committing a crime and it is necessary to reduce the negative consequences of envy for creating healthy organizations. When employees demonstrate the behavior of instigated incivility and reduced involvement in the sequential presence of resource depletion, control efforts in the form of meaningful work are required. At work, meaningful work plays an important role in moderating the effect of the span of supervision on envy, and to our knowledge, this is the first empirical test examining the role of meaningful work as a significant moderator in the relationship between span of supervision and envy. Further analysis of complexities associated with the existing model would require bolt-on work by incorporating various specific parameters. This study contributes to the literature and can serve as a foundation for future research into workplace envy. Thus, the novel findings provide notable insights and paint a more comprehensive picture of the antecedents and consequences of envy.

## Limitations and Future Directions

One of the limitations of the current research study starts with the basic and primary elements of the research. Since this research study is based on the data collected from the food industry of Pakistan listed with the stock exchange, it may limit the scope to a particular context. Envy as a severe threat to organizations should be studied in other organizational and regional contexts. Moreover, the study can also be conducted on non-profit organizations to implicate the review in different contexts.

In the processes of theory building and concept improvement, the space for the developments always remains open. Since envy has been acknowledged as an interpersonal phenomenon in the current study setting, it has addressed only the behavioral outcomes of envy from envious parties. However, in future researches, it would be helpful to study the experience of being envied, and how it affects the behaviors. Furthermore, the investigation has explored the results associated with coveting, as if it may be a cause of personal strain. In this context, the current study has suggested minimizing the level of envy only by meaningful work. However, some other relevant constructs have yet to be explored, which may help further reduce the uncongenial series of dénouements created by envy. Future studies can examine whether different types of envy exist and explore their potential antecedents and consequences. Furthermore, qualitative nature, where the reasons for envy could be studied, may be another future research activity.

## Data Availability Statement

The original contributions presented in the study are included in the article/Supplementary Material, further inquiries can be directed to the corresponding author.

## Ethics Statement

The studies involving human participants were reviewed and approved by the Ethical Review Committee of Namal. The patients/participants provided their written informed consent to participate in this study.

## Author Contributions

HT, AM, and AAh contributed to the conception and design of the study. MK organized the database. SM performed the statistical analysis. HT wrote the first draft of the manuscript. AAA, EM, AM, and AAh wrote sections of the manuscript. All authors contributed to manuscript revision, read, and approved the submitted version.

## Conflict of Interest

The authors declare that the research was conducted in the absence of any commercial or financial relationships that could be construed as a potential conflict of interest.

## Publisher’s Note

All claims expressed in this article are solely those of the authors and do not necessarily represent those of their affiliated organizations, or those of the publisher, the editors and the reviewers. Any product that may be evaluated in this article, or claim that may be made by its manufacturer, is not guaranteed or endorsed by the publisher.
