# Supplementary material for: Span of Supervision and Repercussions of Envy: The Moderating Role of Meaningful Work
Source: Front Psychol. 2022 Jan 4;12:774688. doi: 10.3389/fpsyg.2021.774688 (PMC8764195; doi:10.3389/fpsyg.2021.774688)
Supplement: Supplementary file 1 [file Data_Sheet_1.docx]

# Appendix

**Span of Supervision**

1. Leaders reported the number of their subordinates in each workgroup.

**Envy**

1. My Supervisor values the efforts of others more than she/he values my efforts.
2. It is somewhat annoying to see others have all the luck in getting the best assignments.
3. I don’t know why, but I usually seem to be the underdog at work.
4. I don’t imagine I’ll ever have a job as good as some that I’ve seen.
5. Most of my coworkers have it better than I do.

**Resource Depletion**

1. I feel mentally exhausted.
2. Right now, it would take a lot of effort for me to concentrate on something.
3. I need something pleasant to make me feel better.
4. I feel motivated.
5. If I were given a difficult task right now, I would give up easily.
6. I feel drained.
7. I have lots of energy.
8. I feel worn out.
9. If I were tempted by something right now, it would be difficult to resist.
10. I would want to quit any difficult task I was given.
11. I feel calm and rational.
12. I can’t absorb any information.
13. I feel lazy.
14. Right now, I would find it difficult to plan ahead.
15. I feel sharp and focused. 16. I want to give up.
16. I want to give up.
17. This would be a good time for me to make an important decision.
18. I feel like my willpower is gone.
19. My mind feels unfocused right now.
20. I feel ready to concentrate.
21. My mental energy is running low.
22. A new challenge would appeal to me right now.
23. I wish I could just relax for a while.
24. I am having a hard time controlling my urges
25. I feel discouraged.

**Work Engagement**

1. I work with intensity on my job.
2. I exert my full effort to my job.
3. I devote a lot of energy to my job.
4. I try my hardest to perform well on my job.
5. I strive as hard as I can to complete my job.
6. I exert a lot of energy on my job.
7. I am enthusiastic about my job.
8. I feel energetic at my job.
9. I am interested in my job.
10. I am proud of my job.
11. I feel positive about my job.
12. I am excited about my job.
13. At work, my mind is focused on my job.
14. At work, I pay a lot of attention to my job.
15. At work, I focus a great deal of attention on my job.
16. At work, I am absorbed by my job.
17. At work, I concentrate on my job.
18. At work, I devote a lot of attention to my job.

**Instigated Incivility**

1. Put you down or was condescending to you
2. Paid little attention to your statement or showed little interest in your opinion
3. Made demeaning or derogatory remarks about you
4. Addressed you in unprofessional terms, either publicly or privately

**Meaningful Work**

1. I have found a meaningful career.
2. I understand how my work contributes to my life’s meaning.
3. I have a good sense of what makes my job meaningful
4. I have discovered work that has a satisfying purpose.
5. I view my work as contributing to my personal growth
6. My work helps me better understand myself.
7. My work helps me make sense of the world around me.
8. My work really makes no difference to the world.
9. I know my work makes a positive difference in the world.
10. The work I do serves a greater purpose.
